# Prevalence and outcomes of breast milk expressing in women with healthy term infants: a systematic review

**DOI:** 10.1186/1471-2393-13-212

**Published:** 2013-11-19

**Authors:** Helene M Johns, Della A Forster, Lisa H Amir, Helen L McLachlan

**Affiliations:** 1 Mother & Child Health Research, La Trobe University, Melbourne, Victoria, Australia; 2 Royal Women’s Hospital, Parkville, Victoria, Australia; 3 School of Nursing and Midwifery, La Trobe University, Bundoora, Victoria, Australia

## Abstract

**Background:**

Expressing breast milk has become increasingly prevalent, particularly in some developed countries. Concurrently, breast pumps have evolved to be more sophisticated and aesthetically appealing, adapted for domestic use, and have become more readily available. In the past, expressed breast milk feeding was predominantly for those infants who were premature, small or unwell; however it has become increasingly common for healthy term infants. The aim of this paper is to systematically explore the literature related to breast milk expressing by women who have healthy term infants, including the prevalence of breast milk expressing, reported reasons for, methods of, and outcomes related to, expressing.

**Methods:**

Databases (Medline, CINAHL, JSTOR, ProQuest Central, PsycINFO, PubMed and the Cochrane library) were searched using the keywords milk expression, breast milk expression, breast milk pumping, prevalence, outcomes, statistics and data, with no limit on year of publication. Reference lists of identified papers were also examined. A hand-search was conducted at the Australian Breastfeeding Association Lactation Resource Centre. Only English language papers were included. All papers about expressing breast milk for healthy term infants were considered for inclusion, with a focus on the prevalence, methods, reasons for and outcomes of breast milk expression.

**Results:**

A total of twenty two papers were relevant to breast milk expression, but only seven papers reported the prevalence and/or outcomes of expressing amongst mothers of well term infants; all of the identified papers were published between 1999 and 2012. Many were descriptive rather than analytical and some were commentaries which included calls for more research, more dialogue and clearer definitions of breastfeeding. While some studies found an association between expressing and the success and duration of breastfeeding, others found the opposite. In some cases these inconsistencies were compounded by imprecise definitions of breastfeeding and breast milk feeding.

**Conclusions:**

There is limited evidence about the prevalence and outcomes of expressing breast milk amongst mothers of healthy term infants. The practice of expressing breast milk has increased along with the commercial availability of a range of infant feeding equipment. The reasons for expressing have become more complex while the outcomes, when they have been examined, are contradictory.

## Background

Although data are collected about the proportion of women breastfeeding on discharge from hospital, little is known about how many women are expressing to provide breast milk feeds in addition to, or as an alternative, to feeding directly at the breast. There has been some discussion about increasing numbers of women in Australia, United States of America, the United Kingdom and Singapore expressing to give breast milk feeds rather than breastfeeding directly from the breast [1-6]. Only two studies, one conducted in Australia and one in Singapore [2,6], measured expressing over time. Both reported an increase [2,6].

From an historical point of view, Fildes’ 1986 publication about the history of infant feeding provides a comprehensive insight into infant feeding practices from antiquity and describes related medical practices, popular customs and beliefs [7]. The ‘drawing off’ of breast milk was discussed by Avicenna (AD 980–1036) in the context of milk that was believed to be unpleasant smelling or too thick for the baby to drink [7]. Subsequent references to expressing describe the sucking glass, first mentioned in the mid-16th century [7,8]. The mother applied a glass cup to her breast and sucked on the end of its long glass stem to express milk when her nipples were cracked, or her breast inflamed or infected. During a time when there was concern about the undesirable effects of feeding colostrum to the newborn in pre-industrial Europe, the sucking glass was used as an alternative to employing children or puppies to remove this early milk while the baby was fed by a wet nurse [7].

Developments in breast pump design and uptake over the last century are reflected in changes in ‘brand’ or company names during the same period. A collection at the Powerhouse Museum in Sydney, Australia includes the *Breast Exhauster* (1892), the *Breast Reliever* (1947), the more recent *Kaneson* hand pump (1973) [9] and the water operated *Ellis Expressor* (1970), locally designed to be connected to a kitchen tap [10]. By the early 1980s, breast pumps were transformed, as the red rubber tubing and glass apparatus and, in the case of the electric breast pump, the noisy motor, were substituted for more appealing designs. Pastel colours, discreet motors and less angular shapes became the norm and these pumps are now promoted with names that are arguably designed to enhance market acceptability. In addition to those mentioned in the previous paragraph, examples in name and design are seen in the earlier *Lopuco* and *Egnell electric*[11] and their successors, the *Diana, Freestyle, Pump-in-style, Symphony, Swing, Harmony, Elite* and *Purely Yours* pumps [12,13].

In the world of parenting print media, breast pumps have a growing advertising presence. A hand search of the catalogue of *Essence*, the bi-monthly member magazine of the Australian Breastfeeding Association (ABA) demonstrates a change in the focus of consumer discussion about breastfeeding over time. Breastfeeding is convenient, and advertising for breast pumps may be interpreted as suggesting that expressing is equally so. Blum writes of “the new regularized, fetishized breastfeeding . . . exemplified in the widespread advertising of pumps” [14] (p. 55). Breast milk expression appears to have become more popular as the associated equipment has become more sophisticated and readily available.

Many of the studies about expressing breast milk focus on premature and/or unwell infants [15-17] reflecting the main reasons women expressed to feed their infants in the past. It is likely that up until the last 20 years healthy term infants were either breastfed or bottle fed with infant formula. Although more recent literature has discussed the prevalence of breast milk expression and suggested that more women are expressing their milk [1,5], measurement of this phenomenon is limited and the consequences relatively unknown.

Defining breastfeeding is complex. Discussion has previously focused on the accurate measurement of breast milk feeding; its exclusivity and duration [18]. That is, breastfeeding was the term used to describe any breast milk intake regardless of the mode of its delivery. The focus of recent debate has shifted and the emerging popularity of expressing presents another complexity; the need to find out how breast milk is given, directly at the breast, or otherwise [19]. In addition, Geraghty and Rasmussen have recommended a need to identify at what age the infant is exposed to expressed breast milk, and whose milk is being used [20].

In this paper *expressing (*also known as *pumping*) is used to describe using a pump to obtain breast milk, and *hand expressing* is used for instances where expressing is done by hand. *Breastfeeding* is used to describe the act of feeding directly from the breast, and *breast milk feeding* includes any means by which breast milk is given to the infant.

The aim of this paper is to systematically explore the literature related to breast milk expressing by women who have healthy term infants, including the prevalence of breast milk expressing, and the reported reasons for, methods of, and outcomes related to expressing.

## Methods

The literature search for this paper included a search of Medline, CINAHL, JSTOR, ProQuest Central, PsycINFO, PubMed databases and the Cochrane library with no limit on the year of publication. Reference lists of identified papers were also examined. A hand search of consumer-focused breastfeeding newsletters was undertaken at the ABA Lactation Resource Centre in Melbourne, Australia which holds a collection of more than 18,000 documents related to human lactation. Relevant media and conference proceedings and personal communications were also searched. Only English language papers were included. Keywords used were: milk expression, breast milk expression, breast milk pumping, prevalence, outcomes, statistics and data. The date of the most recent electronic search was 26 February 2013.

Most of the articles identified in the search were specifically about expressing breast milk for sick and/or premature infants and therefore not relevant for this review, however these groups were included in the original search to ensure all relevant articles were located. A flowchart was developed according to PRISMA guidelines to summarise articles obtained in the literature search [21]. This tool is used to illustrate how many references have been located, the number of exclusions and the criteria for and number of eventual inclusions in the completed review.

All papers about expressing breast milk for healthy term infants were considered for inclusion. Papers about expressing that focused exclusively on premature infants were not included except where relevant for other aspects of this literature review (eg, Methods of expressing).

In the Results, the literature has been classified according to: prevalence of breast milk expressing, methods of expressing and reasons why women express. In addition outcomes and other implications of expressing are included. In each section the type and quality of papers identified is described and the papers are summarised and presented in tables under topic headings.

## Results

**Figure 1:**
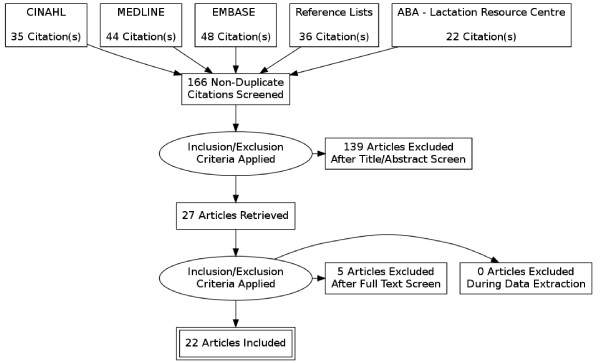
Database search.

A total of 22 papers identified were relevant to breast milk expression, but only seven papers reported the prevalence and/or outcomes of expressing amongst mothers of well term infants. Figure 1 provides a visual representation of the publications identified and reviewed [21]. All of the included papers were published subsequent to 1999. Many papers were descriptive rather than analytical and some were commentaries [22-24], including calls for more research, more dialogue and clearer definitions of infant feeding practice [20,22,25,26].

Included papers are summarised in tables, in chronological order, under the sub-headings:

• Prevalence of breast milk expressing;

• Methods of expressing;

• Reasons why women express their milk;

• Impact of expressing on breastfeeding outcomes;

• Other implications of expressing.

### Prevalence of breast milk expressing

Seven papers were identified that reported on the prevalence of expressing amongst mothers of well, term infants. They came mainly from USA and Australia [1-6,27]. The papers vary in quality and design (Table 1).

**Table 1:** Studies exploring prevalence of breast milk expressing

**Author, year, country**	**Design**	**Location, participants, year of study and recruitment**	**Study aims and outcome measures**	**Results**	**Strengths/*Limitations***
**Geraghty et al. 2005** USA [1]	Retrospective cohort	Cincinnati, Ohio n = 346 2002 Random selection postal recruitment when infants were between 2 and 3 years old	Quantify breast pump use	77% (182/236) ever used a breast pump	Large sample size
			Identify relationships between breast pump use and	No significant difference in expressing between 4 groups of mothers; those of	Clear differentiation between breastfeeding and breast milk feeding
			- singleton vs. multiple pregnancy	- term singletons	Periodic reports re. proportion of expressing versus breastfeeding (at 1 day, 3 days, 2 weeks and monthly until 6 months)
			- gestation at birth	- preterm singletons	*Retrospective data, possible recall bias, initial contact made when children between 2–3 years of age-requesting detailed information about infant feeding at 24 hrs, 3 days, 2 weeks and then at monthly intervals to 6 months.*
			- breastfeeding outcomes	- term multiples	*Expressing methods not distinguished between hand, manual or electric pump*
				- preterm multiples	
				- 68% (236/346) received at least some breast milk.	
				- 5% (12/236) exclusively expressed to feed – all mothers of premature babies	
**Binns et al. 2006** Australia [2]	Longitudinal	Perth, Western Australia	- Explore determinants of breastfeeding	- PIFS I 38% (211/556) expressing by 6 weeks	Large sample
		PIFS I n = 556	- Measure and compare prevalence of expressing	- PIFS II 69% (405/587) expressing by 4 weeks	Comparison of similar groups 10 years apart
		1992–93		- Expressing rates steadily decline after 6 weeks:	*Limited detail about expressed breast milk/ breastfeeding proportions.*
		PIFS II n = 587		26% (145/556) at 24 weeks in PIFS I 28% (164/587) at 22 weeks in PIFS II	*Public patients only, perhaps not representative*
		2002–03			*Differing interview schedules – less clarity for comparison*
		Recruited in hospital in early postpartum period			
**Labiner-Wolfe et al. 2008** USA [3]	Longitudinal	National study	- Reasons why women express	- Most common reason: for someone else to feed baby	Large sample
		n = 4606	- Amount and prevalence of milk expression	- 85% (1329 /1564) between 1.5 and 4.5 months postpartum had expressed at some time since birth	Maternal recall previous 7 days
		2005–2007	- Associated socio-demographic factors	- 68% (1015/1493) of this group had expressed in 2 weeks before survey, 25% (373/1493) regularly	Measures frequency of expressing (asked how many times expressed in previous 2 weeks and if expressed on a regular schedule)
		from IFPS II		- Expressing associated with: maternal employment, higher income, first breastfeeding experience	*Not nationally representative: Older, more educated, more likely to be white, employed, higher income, less likely to smoke. More likely to breastfeed and for longer*
		Mail survey 2, 5 and 7 months postpartum			
**Shealy et al. 2008** USA [4]	Longitudinal	National study	Describe breastfeeding in first 12 months to identify:	- 0.06% of babies fed expressed breast milk exclusively – 2/3 of these ceased breast milk feeding by 4 weeks	Detailed analysis of feeding type/frequency/duration of individual feeds
		n = 2587	- Prevalence of exclusive pumping and formula supplementing		*Limited data re. expressing – except when it was exclusive*
		2005–2007	- Patterns and trends in breastfeeding related to common advice given		
		from IFPS II			
		Monthly postal questionnaires			
**Clemons & Amir 2010** Australia [5]	Cross-sectional	State-wide study, Victoria	- Prevalence of breast milk expression	- 67% (602/898) had fully breastfed prior to hospital discharge	Large study
		n = 903	- Demographic characteristics of women who express, why and how they do it	- 14% (125/898) had breastfed and expressed to feed their baby	*Possible selection bias (members of the Australian Breastfeeding Association)*
		2008	- Women’s experience of using breast pumps	- Of those whose youngest child was over six months 95% (628/661) fully breastfed for at least six months	*Timing of questionnaire, possible recall bias*
		Online questionnaire sent to Australian Breastfeeding Association members who had an email address		- 4% (34/898) expressed and exclusively fed EBM	
		Baby any age		- 98% (885/903) ever expressed	
**Hornbeak et al. 2010** Singapore [6]	Retrospective cohort	Singapore	- Prevalence and patterns of breastfeeding in Singaporean Chinese mothers from birth to 6 months	- Initiation of breast milk feeding increased from 69% (144/210) in 2000-2001 to 82% (538/656) in 2006/2008	Large representative sample of Chinese Singaporean mothers
		n = 3009		- Exclusive expressing increased from 9% (18/144) in 2000-2001 to 18% (118/538) in 2006/2008	*Limited detail about expressed breast milk/breastfeeding proportions.*
		2006-2008		- Direct breast milk feeding decreased from 34% (72/210) in 2000–2001 to 27% (142/656) in 2006/2008	*Possible recall bias - recruitment 6–72 months after birth*
		Recruited mothers of 6-72 month Chinese Singaporean children through Strabismus, Amblyopia and Refractive Error in Singaporean Children (STARS) Study			*Gestational age not indicated*
		Mailed invitation			
		Face-to-face interview			
**Geraghty et al. 2012** USA [29]	Prospective longitudinal cohort	Cincinnati, Ohio	- Describe who commences expressing early	- 14% (8/59) commenced some expressing in first week	Prospective design assisting recall
		n = 60	- Explore breastfeeding duration in women who express	- By four weeks: 63% (37/59) expressing	Initial weekly visits; used weekly and 24 hour recall to enquire about feeding and expressing
		2004–2007		- Expressing had no effect on duration of breast milk feeding	Clear differentiation between ‘breastfeeding’ and ‘breast milk feeding’
		Recruited face to face in first week after hospital discharge.			*Small study*
					*Recruitment of women who planned to breastfeed for 6 months or more*
					*Mothers recruited for study knew they were going to be assisted to pump and may have been more likely to be comfortable with this.*
					*Possible introduction of bias as weekly collection of breast milk was initiated at 1 week by research nurse using an electric breast pump*

Although several papers refer to an *increase* in the prevalence of expressing, the only data that actually documented such an increase were from Western Australia and Singapore [2,6]. The Perth Infant Feeding Study I (PIFS I), conducted in 1992–93 was followed by the Perth Infant Feeding Study II (PIFS II) ten years later [2]. Binns et al. reported the proportion of mothers who expressed breast milk (not necessarily expressing exclusively) during the first six weeks after birth, and found a 31% increase over ten years as well as a gradual decline in expressing after the first six weeks [2]. (The Singapore study reports exclusive expressing and is discussed below [6]).

In Mid-Western USA in 2002, Geraghty et al. found only sixteen percent (55/346) of women breastfed exclusively at the breast for the duration of their lactation and just seven percent (24/346) of the same group breastfed exclusively at the breast for a minimum of six months [1]. The authors concluded that expressing had become an integral part of human milk feeding [1]. Also in the USA, Labiner-Wolfe et al. analysed data from the Infant Feeding Practices Study II (IFPS II) (collected between 2005 and 2007), and found that 85% (1329/1564) of breastfeeding women had expressed breast milk, and that of these women, more than half had done so in the first week after birth [3]. An online study in Victoria, Australia found that 98% (885/903) of breastfeeding women had expressed at some time [5], however, this was a very select group – as respondents were all members of the ABA.

A small proportion of women never breastfeed, but rather exclusively breast milk feed using expressed breast milk. Geraghty et al. reported that five percent of women exclusively expressed and fed breast milk, all of whom were mothers of premature infants [1]. Another study in the USA identified a six percent exclusive expressing rate amongst infants between 35 and 45 weeks gestation [4]. In Australia, Clemons and Amir found that four percent of women in their cross-sectional study expressed exclusively [5]. In comparison, the exclusive expressing group was higher in Singapore; increasing from nine to eighteen percent between 2000–01 and 2006–08, apparently at the expense of direct breastfeeding which decreased from 34 to 22 percent over the same time period [6].

### Methods of expressing

A Cochrane review, which included 23 studies assessing breast milk expression methods found that there was no difference between manual and electric pumps in terms of breast milk production [28]. Most of the studies in the systematic review were excluded from this review because they did not meet inclusion criteria, mainly because they focused on premature or unwell infants. The papers discussed in this section are described in Table 2.

**Table 2:** Studies exploring methods of expressing

**Author, year, country**	**Design**	**Location, participants, year of study and recruitment**	**Study aims and outcome measures**	**Results**	**Strengths/Limitations**
**Binns et al. 2006** Australia [2]	Longitudinal	Perth, Western Australia	- Explore determinants of breastfeeding	- Approx. 60% (n = 1143) using manual pumps in both studies	Large study
		PIFS I n = 556	- Measure and compare prevalence of expressing 1992-93 and 2002-03	- Use of electric pumps increased by 31% in 10 years	Comparison of similar groups 10 years apart
		1992–93			*Actual figures not given*
		PIFS II n = 587			*Public patients only, perhaps not representative*
		2002–03			
		Recruited in hospital in early post-partum period			
**Labiner-Wolfe et al. 2008** USA [3]	Longitudinal	National study	- Reasons why women express	**- Birth to 1.5 -4.5 months**	Large sample size
		n = 4606	- Amount and prevalence of milk expression	80% (105/1302) battery or electric 44% (573/1302) manual pump 14% (18/1302) hand	3 mailed questionnaires seeking information re. feeding in previous fortnight - recall bias unlikely
		2005-2007 IFPS II	- Associated socio-demographic factors	**- Previous 3 months to 6.5-9.5 months**	Detailed information re. methods of expression over time
		Mail survey 2, 5 and 7 months postpartum		73% (39/529) battery or electric) 33% (18/529) manual pump 13% (69/529) hand	*Not nationally representative, older, more educated, more likely to be white, employed, higher income, less likely to smoke. More likely to breastfeed and for longer*
**Ohyama et al 2010** Japan [31]	Sequential crossover	Yokohama, Kanagawa	- Comparison of effectiveness and comfort of manual and electric breast expression in first 48 hours after birth	- Manual expressing associated with greater milk volume: net milk yield per woman 2 ml.	Limited other exploration of this area
		n = 11		- Manual expression 2 ml (median; range: 0-12.6 ml.)	*Small study*
		2003-2004		- Electric expression 0.6 ml. (0-7.2 ml.) (P < 0.05).	*Infant gestation and health status not indicated*
		Mothers of infants admitted to neonatal intensive care recruited in hospital soon after birth		- Manual pump associated with more reports of pain	
**Flaherman et al 2012** USA [32]	RCT	San Francisco & Sacramento, California	Comparison of hand and electric expression measured;	- At 2 months mothers assigned to hand expressing were more likely to be breastfeeding (97%, 47/48) than mothers assigned to breast pumping (73%,35/48) (RR:1.32, 95% CI 1.01,1.73)	Limited other exploration of this area, no previous studies linking type of expressing to breastfeeding outcomes
		n = 68	- Milk transfer		Thorough discussion
		2007-2009	- Breast pain		*Small study, final outcome assessment based on 48 participants*
		Recruited12-36 hours after birth	- Breastfeeding confidence		*Possible bias- recruited infants experiencing feeding difficulty*
			- Breast milk expression experience		*No control group*
			- Breastfeeding rates at 2 months		
**Becker et al. 2011** UK [30]	Systematic review	International	- Assessment and review of randomised and quasi randomised trials comparing methods of milk expression any time after birth and crossover trials commencing at least 28 days after birth	- More milk with relaxation tape	Systematic review
		n = 642 women from 23 studies		- No difference in mean vol. with simultaneous or sequential pumping, or between manual and electric pumps studied	*Most studies specifically related to the care of the pre-term infant*

Given its universal accessibility, the simplest way to express milk is by hand, and evidence from a Japanese study demonstrates that this is the most effective method to use when expressing colostrum [29]. Hand expressing however, was associated with increased reports of local pain compared to electric breast pumping [29]. A randomised trial in the USA which compared hand expressing and pump use found that early hand expression appeared to improve breastfeeding rates at two months when compared with using a pump [30].

Although hand expression may be just as effective [28], and electric breast pumps are considerably more expensive than simple hand expression or the use of a hand operated pump, the use of electric breast pumps has become more popular over the last decade [31], and in Perth, Australia, has increased threefold in ten years [2]. Electric breast pumps are a regular feature of postnatal wards of maternity hospitals in Washington, DC where Buckley, examining the views of lactation consultants about breast pumps, writes about the prominence of the breast pump, evidenced by the universality of its provision, in her words, “*A breast pump for every room*” [31] (p.16). The accessibility of electric breast pumps is demonstrated in an online study in Australia which found that 66% (556/843) of breastfeeding women had used an electric breast pump [5]. In a mail survey of 3,606 women from the IFPS II in the USA, women who undertook regular scheduled expressing were more likely to use electric breast pumps [3].

### Reasons why women express their milk

Factors that appear to be associated with women’s decisions to express their breast milk have been reported in some papers (Table 3). Women who experience difficulty establishing breastfeeding are more likely to express [2,3,5,27], and mothers with premature or low birth weight infants, mothers who are unwell, those who have not previously breastfed are also more likely to express [5,27].

**Table 3:** Studies exploring reasons women express

**Author, year, country**	**Design**	**Location, participants, year of study and recruitment**	**Study aims and outcome measures**	**Results**	**Strengths/Limitations**
**Dykes & Williams 1999** UK [34]	Longitudinal, phenomenological study	Northern England,	- Explore women’s experience of expressing particularly perception of adequacy of milk supply	- Beliefs re. adequacy of breast milk supply influenced by interplay of feeding management, infant behaviour, lactation physiology and maternal mental health.	*Small mono-cultural group*
		n = 10			
		1998			
		Postnatal primiparas recruited face-to-face in hospital, home visits at 6, 8 &12 weeks			
**Binns et al. 2006** Australia [2]	Longitudinal cohort	Perth, Western Australia	- Explore determinants of breastfeeding	- Early breastfeeding difficulties,	Comparison of similar groups 10 years apart
		PIFS I n = 556	- Measure and compare prevalence in expressing	- Engorgement, sore nipples, mastitis	*Mainly women who expressed to manage breastfeeding difficulties*
		1992–93		- Feed to be given by someone else	*Public patients only, perhaps not representative*
		PIFS II n = 587		- To store extra milk	
		2002–03		- Father to feed	
		Recruited in hospital in early post-partum period.		- To increase supply	
				- Feeding/attachment problems	
				- To get baby to drink from a bottle	
				- Just to try it out	
**Labiner-Wolfe et al. 2008** USA [3]	Longitudinal cohort	National study	- Reasons why women express	- to allow someone else to feed	Large sample
		n = 3606	- Amount and prevalence of milk expression	- maternal employment	*Not nationally representative Participants older, more likely to be educated, white, employed, higher income*
		2005–2007	- Associated socio-demographic factors	- to have an emergency milk supply	
		from IFPS II		- no previous breastfeeding experience	
				- geographic location (Midwest Vs. West)	
				- embarrassed to breastfeed in public	
**Buckley 2009** USA [33]	Focus groups	Washington, DC	- Ascertain lactation consultant’s beliefs and experiences re. impact of breast pumps on breastfeeding practice	- Technological birth contributes to technological breastfeeding	Exploration of professional attitudes to change in feeding practice -no previous exploration of this area
		n = 12		- Engorgement, plugged ducts, to increase supply, to stimulate the let-down reflex, to pull out inverted nipples.	*Small sample size*
		Lactation consultants		- Return to work	*Volunteer participants*
		Purposeful sampling		- Measuring milk, diminished confidence in ability to provide enough milk	*Date of study not indicated*
**Clemons & Amir 2010** Australia [5]	Cross-sectional	State-wide study, Victoria	- Prevalence of breast milk expression	- Premature baby/sick mother or baby	Large study
		n = 903	- Demographic characteristics of women who express, why and how they do it	- Attachment problems/not drinking well	*Possible selection bias (members of ABA)*
		2008	- Women’s experience of using breast pumps	- Advised	*Timing of questionnaire, possible recall bias*
		Online questionnaire sent to Australian Breastfeeding Association (ABA) members who had an email address		- Not enough milk/To store extra milk	
				- Nipple pain	
				- Engorged breasts/mastitis	
				- So someone else can feed baby	
				- Maternal work	
				- Just to try it out	
				- To allow mother to drink alcohol	
				- Uncomfortable breastfeeding in public	
**Geraghty et al. 2012** USA [29]	Prospective longitudinal cohort	Cincinnati	- Duration of breast milk feeding	- Planned return to work by 6 months	Prospective design
		n = 60	- Describe who commences expressing early		*Small study*
		2004–2007			*Recruitment of women who planned to breastfeed for 6 months or more*
		recruited face to face			*Mothers recruited for study knew they were going to be assisted to pump and may have been more likely to be comfortable with this.*
					*Possible introduction of bias as weekly collection of breast milk was initiated at 1 week by research nurse using an electric breast pump*

Women with an elevated body mass index (BMI) are more likely to express their milk than to breastfeed, perhaps related to anxiety about exposing their bodies [27,32]. Obese women often have large breasts and may experience difficulty feeding discretely [33]. In addition these women may express because of physical difficulty with breastfeeding. Large breast size may impede maternal ability to see and or facilitate appropriate infant attachment and feeding [33]. Leonard et al. investigated breast milk expressing behaviours and concluded that expressing may support longer durations of breastfeeding in overweight or obese women [32]. Embarrassment about breastfeeding in public has been identified as a reason women express regardless of cultural background or body size [5,34,35]. Cultural differences may inhibit women from breastfeeding outside the home, leading to some women expressing so that they can avoid exposing their bodies in public [3,5,34].

Other reasons women express include breastfeeding problems such as mastitis and breast engorgement [2]; nipple pain and difficulty with attachment to the breast [5]; concern about oversupply or undersupply [3,5,34,36] and allowing the baby to be fed by someone other than his/her mother [2,3,34,37]. Women express in order to return to paid work [3,5,38]. Those who are in paid employment are more likely to express their milk when there are flexible work arrangements and designated places to express [39,40]. In addition to women who are in paid employment expressing milk, women who have a high income are also more likely to do so [3].

### Impact of expressing on breastfeeding outcomes

There have been contradictory reports regarding the association between expressing breast milk and the success and duration of breastfeeding (Table 4). Some studies suggest that expressing to feed (as opposed to breastfeeding solely at the breast) in the early postpartum period is associated with shorter duration of breastfeeding [1,41], while others have found the reverse [42,43]. Binns et al. report on trends in the expression of breastmilk and conclude “The appropriate use of expressed breastmilk allows a mother to achieve six months of exclusive breastfeeding while giving her more options with regards to paid work or study and the management of breastfeeding difficulties”[2] (Page 8). Women who fed at the breast only were found by Schwartz et al. to breastfeed for longer [41] and Chapman et al. reported that expressing did not improve milk volumes or duration of breastfeeding [44]. On the other hand, Win et al. in Perth, Australia, explored the association between expressing and the duration of breastfeeding and reported that mothers who expressed were more likely to be breastfeeding at six months than those who didn’t express [45].

**Table 4:** Impact of expressing on breastfeeding outcomes

**Author, year, country**	**Design**	**Location, participants, year of study and recruitment**	**Study aims and outcome measures**	**Results**	**Strengths/Limitations**
**Chapman et al. 2001** USA [45]	RCT	Hartford, Connecticut	Effects of expressing before the onset of lactation :	- No significant difference in milk transfer or breastfeeding duration between women who expressed breast milk and those who did not.	*Only women who had a caesarean section*
		n = 60	- on early milk transfer	- Primiparous women in pumping group breastfed for 5 months less than those in control group but this finding was not statistically significant.	*Study underpowered for primiparous women*
		1997–1998	- on subsequent breastfeeding duration		
		Convenience sample 8–24 hours post Caesarean Section			
**Schwartz et al. 2002** USA [42]	Prospective cohort	Detroit, Ann Arbor and Southfield, Michigan and Omaha, Nebraska	- Determine demographic, behavioural and clinical factors associated with weaning from breast in the first 12 weeks	- Michigan women (n = 711) who expressed breast milk were 3 times more likely to wean than those who didn’t (Hazard Ratio: 3.0 95% CI 1.3,6.7)	Large study
		n = 946		- Nebraska women (n = 235) showed no association between pumping and weaning (HR: 0.6, 95% CI 0.3,1.5)	*Only measured to 12 weeks*
		1994–1998			*Non-representative sample*
		Recruitment:			*Michigan group were recruited from an alternative birthing centre and were significantly more likely to be older than 30 years, have a bachelor’s degree, have 3 or more children and have had a vaginal birth*
		Michigan - at birth centre orientation			
		Nebraska - on maternity leave application to large company			
**Ortiz 2004** USA [37]	Clinical audit	Burbank, California	- Duration of breast milk feeding related to a range of employee chosen lactation support options	- 98% (452/ 462) breastfeeding initiation	Large study over 4.5 years
		n = 462		- 74% (246/332) expressed milk until infant at least 6 months	*Limited differentiation between breastfeeding and expressing / breast milk feeding*
		1993–1999		- 24% (81/332) expressed milk until infant at least 12 months	*No information re any other infant feeding/exclusivity of breast milk feeding*
		Antenatal recruitment in workplace		- Mean age of infants at maternal cessation of pumping at work 6.3 months	*No consideration of options in the workplace to breastfeed at the breast*
**Geraghty et al. 2005** USA [1]	Retrospective cohort	Cincinnati, Ohio	Measure breast pump use	Of breast milk feeding mothers:	Large sample size
		n = 346	Identify relationships between breast pump use and:	- 10% (24/346) breastfed exclusively for a minimum of 6 months	Breastfeeding / breast milk feeding clearly differentiated
		2002	- singleton vs. multiple pregnancy	- 16% (55/346) breastfed exclusively for duration of their breast milk feeding	Periodic reports re. proportion of expressing versus breastfeeding (at 1 day, 3 days, 2 weeks and monthly until 6 months)
		Random selection Postal recruitment when infants were between 2 and 3 years old	- gestation at birth	- 77% (182/236) expressed at some time in first 6 months	*Retrospective data, possible recall bias as participants were recruited 2 or more years post birth*
			- breastfeeding outcomes	- 59% (140/236 ) ceased breast milk feeding by 6 months	
				Of the 140 women who had ceased breast milk feeding by 6 months, at the time point just prior to exclusive formula feeding:	
				- 76% (106/140) were either expressing exclusively or combining expressing with breastfeeding	
				- 24% (34/140) were breastfeeding	
				Early breastfeeding associated with a longer duration of breast milk feeding	
**Win et al. 2006** Australia [55]	Prospective cohort	Perth, Western Australia	- Investigate association between breast milk expression and breastfeeding duration	- Mothers who expressed at least once more likely to be breastfeeding at 6 months (RR: 0.71, 95% CI 0.52,0.98)	Prospective design assisting recall
		PIFS II			*Ever “expressed” / “any” breastfeeding*
		n = 587			*? lower socio economic bias*
		2002–03			*No account of frequency of expressing*
		Recruited in hospital at birth.			
**Meehan et al. 2008** USA [43]	Quasi-experimental	Los Angeles, California	- Evaluation of program to facilitate breastfeeding for low income mothers	- Electric pump loan associated with more breastfeeding at 6 months. Mothers loaned a breast pump 5.5 times more likely to than those who hadn’t received one to not have requested formula by 6 months	*Limited reliability of proxy measurement to assess breast milk feeding prevalence or duration*
		n = 208	- Maternal request for formula from WIC program used as proxy measurement to give indication of partial breastfeeding	(OR: 5.5, 95% CI 2.0,15.1)	*No differentiation between breastfeeding and breast milk feeding*
		2001			
		Breast pump loan program for low income Women with Children (WIC) recipients			
**Fein 2008** USA [41]	Prospective cohort	National	- Examine strategies used to combine work and breastfeeding	Median duration of breast milk feeding associated with workplace practices:	Large National study
		n = 810	- Identify strategies associated with enhanced breastfeeding intensity/longer duration		Prospective design
		2005–2007		- expressing and breastfeeding (32.4 weeks) (n = 75)	Questionnaire design with 7 day recall
		from IFPS II		- breastfeed at the breast only (31.4 weeks) (n = 250)	*No description of feeding method away from workplace*
		Recruitment via postal questionnaire in late pregnancy		- expressing only (26.3 weeks) (n = 75)	*Older, less educated, low income and women from racial/ethnic minority groups underrepresented*
				- neither breastfeeding or expressing (14.3 weeks) (n = 128)	
**Clemons & Amir 2010** Australia [5]	Cross-sectional	State-wide, Victoria	- Prevalence of breast milk expression	- 27% (218/903) indicated that expressing had allowed them to breastfeed for longer	Large study
		n = 903	- Demographic characteristics of women who express, why and how they do it		*Possible selection bias (members of ABA)*
		2008	- Women’s experience of using breast pumps		*Timing of questionnaire, possible recall bias*
		online questionnaire			
		ABA members with internet addresses			
**Dabritz et al. 2010** USA [56]	Retrospective cohort	Yolo County, California	- Assess relationship between maternal experience in hospital and any breastfeeding at six months	- Almost exclusive breastfeeding at 6 months associated with not using a breast pump in hospital 77% (93/121) compared to 21% (25/121) who did use a pump in hospital (OR: 0.6 95% CI 0.3,1.0)	*Differentiation between breastfeeding and breast milk feeding unclear*
		n = 382			
		2006–07			*Possible recall bias - interviews 6–9 months after birth*
		Recruited in community after birth - 8 months			
**Hornbeak et al. 2010** Singapore [6]	Retrospective cohort	Singapore	- Record prevalence and patterns of breastfeeding in Singaporean Chinese mothers	- Breast milk feeding initiation and duration increased over time and were independently associated with higher maternal education, increased milk expression and complementary feeding	Large representative sample of Chinese Singaporean mothers
		n = 3009		Changes between 2000–01 and 2006–08:	*Possible recall bias - recruitment 6–72 months after birth*
		2006–2008		Infant formula feeding 31% (66/210) to 18% (118/656)	*Gestational age not indicated*
		Recruited mothers of 6–72 month Chinese Singaporean children through STARS		Breast milk feed initiation 69% (144/210) to 82% (538/656)	
		Mailed invitation		Expressed breast milk 9% (18/210) to 18% (118/656)	
				Combination feeding 26% (54/210) to 41% (269/656)	
**Geraghty et al. 2012** USA [29]	Prospective cohort	Cincinnati, Ohio	- Determine who expresses their milk by end of 4 weeks and how long they continue feeding	- Milk expression common in first month postpartum	Prospective design
		n = 60		- Milk expression by 4 weeks did not significantly influence duration of breast milk feeding	Clear differentiation between breastfeeding and breast milk feeding
		2004–2007			*Recruitment of women who planned to breastfeed for 6 months or more*
		Participants enrolled in a research human milk bank recruited at home in first week postpartum			*Mothers recruited for study knew they were going to be assisted to pump and may have been more likely to be comfortable with this.*
					*Possible introduction of bias as weekly collection of breast milk was initiated at 1 week by research nurse using an electric breast pump*

Studies that looked at duration of breast milk feeding have had differing outcomes. Ortiz et al. explored the duration of breast milk expression for women allocated to a lactation program which provided equipment and support for expressing. Findings indicated that these women were more likely to breast milk feed for longer than those who did not receive such support [39]. Geraghty et al. found that mothers who fed solely at the breast, particularly in the early weeks postpartum, were more likely to breast milk feed for longer than women who had combined breastfeeding with expressing [1].

### Implications for maternal health

Breast pain and nipple trauma have been associated with expressing [5,29]; they contribute to maternal discomfort and distress, and nipple trauma is known to be associated with the development of mastitis [46]. While Thorley identifies compromised mother/infant skin-to-skin contact and bonding as a result of expressing [23], Johnson et al. call breast pumping liberating, giving the mother a means to *“ . . . negotiate some independence and manage the demands of breastfeeding”*[34] (p. 900). These authors suggest that expressing may facilitate maternal independence, and give the mother freedom from the demands of her baby [34].

Considering the paucity of discussion in the literature, it appears that the implications of expressing on maternal mental health warrants attention.

### Other implications of expressing

A range of other possible outcomes of breast milk expression bear consideration and can be seen in Table 5. The additional handling involved in the expression, storage and subsequent bottle feeding of expressed milk creates additional risks for infection in the infant, as discussed and illustrated visually by Geraghty [25]. Breast milk, frozen and fed later loses vitamin content, and, like infant formula, it is at risk of contamination, as it is subject to more handling through the process of preparation [47]. Freezing, defrosting and reheating and microwaving all have the potential to compromise milk quality and safety [48,49].

**Table 5:** Other implications of expressing

**Author, year, country**	**Design**	**Location, participants, year of study and recruitment**	**Study aims and outcome measures**	**Results**	**Strengths/*Limitations***
**Clemons & Amir 2010** Australia [5]	Cross sectional	State-wide study, Victoria	- Prevalence of breast milk expression	- 17% (126/737) experienced nipple pain associated with pump use	Large study
		n = 903	- Demographic characteristics of women who express, why and how they do it		*Possible selection bias (members of ABA)*
		2008	- Women’s experience of using breast pumps		*Timing of questionnaire, possible recall bias*
		Online questionnaire sent to Australian Breastfeeding Association (ABA) members who had an email address			
		Baby any age			
**Li et al. 2010** USA [49]	Longitudinal cohort	National study	- Test infant ability to self-regulate intake – compare active sucking (breastfeeding) with passive feeding (EBM via bottle)	- Infants bottle fed early more likely to empty bottle/cup in late infancy	Large national longitudinal study Minimal reporting bias for exposure and outcome – 7 day retrospective recall
		n = 1597	- Complete empting of bottle or cup in late infancy used to indicate self-regulation	- bottle a totally different feeding mode	Multivariate analysis
		2005–2007			*Maternal report of feeding behaviour/bottle emptying - reporting error possible*
		from IFPS II			
**Li et al. 2012**[50]	Longitudinal cohort	USA	- Multi level analysis to estimate weight gain X type of milk & feeding mode at 3,5, 7.and 12	- Among infants fed only breast milk, Breast milk fed infants gained 780g per month in the first year compared with breastfed infants who gained 729g	Large national longitudinal study Minimal reporting bias for exposure and outcome – 7 day retrospective recall
		n = 1899		- Possible association between bottle feeding EBM and increased weight gain	
		IFPS II			
		2005-2007			
**Geraghty et al. 2012** USA [28]	Retrospective cohort	Cincinnati, Ohio	- Examination of methods of maternal expression and infant consumption of breast milk	- All expressed, all babies fed some expressed milk	Limited other exploration of this area
		n = 40		95% (38/40) infants breastfed and EBM	*Small size*
		2008		37% (15/40) fed EBM same day	*Retrospective data collection*
		Outpatients attending breastfeeding clinic, recruited by mail		30% (12/40) fed EBM same week	*Possible recall bias*
				25% (8/40) fed EBM 1 and 4 weeks later	
				13% (5/40) fed EBM more than 4 weeks later	

The infant fed from a bottle, regardless of the type of milk, is deprived of the benefits of self–regulation of intake associated with breastfeeding, which may increase the risk of subsequent childhood obesity [50,51]. Orofacial implications include the risk of dental caries associated with the use of a teat [52] and orthodontic problems associated with not breastfeeding [53].

## Discussion

Although there is some commentary about an increase in breast milk expressing in the literature, actual measurement of the phenomena is quite limited. In addition, expressing breast milk is anecdotally less common in countries where there are more generous maternity leave provisions in terms of length of financial support, but there is limited evidence in the literature to support this. Two studies, one in Australia and the other in Singapore provide the only data actually documenting an increase in expressing over time [2,6]. Other authors discuss the prevalence of expressing and describe a preponderance of the practice [1-6,27]. This suggests that breast milk feeding solely at the breast is actually quite rare, at least in the developed world. Some breast milk expressing deserves consideration as incidental, something a mother might do only on occasion, for example when she needs to go out without her baby, when introducing infant cereal, when the infant is ill or unable to attach or is refusing the breast. We know that expressing breast milk has become more common, clarification of the amount, the proportion and the frequency of expressing and breast milk feeding is necessary before we can properly explore the implications of this relatively unexplained shift away from the breast.

Although concluding remarks in the 2011 Cochrane review identify the relative effectiveness of hand expressing and less expensive breast pumps [28], it is not surprising that regular scheduled expressing is associated with the use of electric breast pumps [3]. Hand expressing barely rates a mention in the literature about expressing breast milk, although breast pump use and ownership are commonly discussed [2,31,54,55]. Thorley describes breast pumps as a substitute for the skill of hand expression [23]. A recent paper from Flaherman et al. reports on an apparent positive effect of hand expressing when compared with bilateral electric pumping [30]. The authors discuss the possibility that hand expressing contributes to less awkwardness or embarrassment for the mother, who is more likely to be comfortable hand expressing than using a pump when others are present [30]. After the establishment of a mature milk supply however, a 2013 review of studies comparing different methods of milk expression [37], identifies several that found electric breast pumping to be more effective than any other method in terms of milk volume obtained [49,56,57]. The let-down reflex, a physiological response that is the process by which milk becomes available for the baby, can be inhibited by stressful situations such as embarrassment [58]. This may also be the case when unfamiliar equipment such as the breast pump is used. Suggestions of unquantifiable benefits associated with teaching hand expressing are made by Morton as she cautions against an “*over-reliance on mechanical interventions*” [59] (p. 276). Maternal confidence may be enhanced by a more relaxed early postpartum experience. Ease with the mechanics of breastfeeding may be fostered for the woman who has had some experience handling her breasts to obtain milk, as she will have done when hand expressing. The confidence attained by the handling and the achievement of actually expressing, as well as the visualisation of her milk may reinforce such confidence as she has expressed simply, with her own hands and without the complication of any additional mechanisation.

Women express breast milk because of doubt about the adequacy of their milk supply [36]. Many do so because of initial difficulties establishing breastfeeding [2,3,5,27]. Maternal return to work has been identified as another reason [3,5,27]. Workplaces which provide options such as on-site childcare, lactation breaks for expressing and/or breastfeeding foster longer term breast milk feeding [60]. Focus on breast expression facilities may however encourage maternal return to the workforce at the cost of other initiatives, or at the cost of broader social change to support women to spend more time with their infants and by implication, breastfeeding. Elevated BMI, cultural differences and embarrassment about breastfeeding in public all contribute to increasing breast milk expression rates [3,5,27,29,32,34].

Simple attribution of cause and effect is probably inadequate in any discussion of expressing and its impact on breastfeeding success and duration. Several authors appear to refer to ‘any’ expressing without specifying relative quantities or proportions of breast milk feeds given, from the breast or otherwise [2,4,6].

Expressing may contribute to a parental focus on the measurement of breast milk. Such quantification of breast milk may undermine confidence about the adequacy of milk supply, and may be reflected in parental anxiety about not knowing how much milk the baby is obtaining when feeding directly from the breast [31].

## Conclusions

This literature review has found limited evidence about the prevalence and outcomes of expressing breast milk amongst mothers of healthy term infants. Authors use a variety of definitions to describe the various infant feeding options, which limits our ability to make conclusions. The practice of expressing breast milk has increased along with the commercial availability of a range of infant feeding equipment. Expressing breast milk has become more common and introduces an opportunity for others to feed the baby. It could be argued that, for some families, breastfeeding has been reduced to a task, which is that of providing milk for the baby, quite possibly even beyond arm’s length, something that anyone can do and potentially at the cost to the special relationship between a mother and her infant. The reasons for expressing have broadened and acquired complexity, while the outcomes of expressing, when they have been examined, are contradictory.

## Competing interests

The authors declare that they have no competing interests.

## Authors’ contributions

HMJ undertook the literature review and wrote the first draft. All authors contributed intellectual input into revisions of the paper. All authors read and approved the final manuscript.

## Pre-publication history

The pre-publication history for this paper can be accessed here:

http://www.biomedcentral.com/1471-2393/13/212/prepub
